# Editorial: Implementation of genomic and epigenomic innovation in clinical cancer diagnostics

**DOI:** 10.3389/fonc.2023.1253630

**Published:** 2023-08-10

**Authors:** Laila C. Schenkel, Anahita Mohseni Meybodi, Shamini Selvarajah

**Affiliations:** ^1^ Molecular Diagnostics Division, London Health Sciences Centre, Department of Pathology and Laboratory Medicine (PaLM), Western University, London, ON, Canada; ^2^ Clinical Laboratory Genetics, University Health Network, Department of Laboratory Medicine and Pathobiology, University of Toronto, Toronto, ON, Canada

**Keywords:** molecular and cytogenetics, epigenetics, cancer genomics, tumor testing, genetic testing, cancer diagnosis

Precision medicine has revolutionized the field of cancer by providing personalized guidance for the diagnosis, prognosis, and treatment of various types of cancers. Key to the success of precision medicine is the integration of tumor genomics and epigenomics into clinical practice.

Genomic testing in oncology has undergone a dramatic transformation in the past two decades, with the integration of targeted ultra-rapid testing modalities and high throughput next generation sequencing (NGS) platforms that enable comprehensive genomic profiling for the clinical management of patients. Furthermore, DNA methylation, a crucial epigenetic modification, has emerged as a valuable biomarker for cancer diagnosis and management as methylation patterns of specific genes can provide insights into tumor behavior and response to therapy.

While the field of cancer genomics continues to advance rapidly in biomarker discovery and technologies, the process of implementing these discoveries into clinical practice can be challenging. Thorough studies and collaborations are essential to ensure the successful integration of these innovations, enabling their benefits to reach patients and improve cancer management.

This Research Topic focuses on showcasing recent advancements in cancer genomics and their integration into clinical practice. By sharing knowledge and fostering collaboration, the aim is to advance the field of tumor genomic testing and facilitate more precise and personalized cancer care. The following summary highlights the studies presented and their contribution to improving cancer diagnostics and patient care.

Clinical laboratories have widely adopted the utilization of NGS panels for solid tumors testing, which can be observed in the study by Bhai et al. In this study targeted NGS assays were utilized to analyze 3164 solid tumor samples over a period of two years. The results demonstrated the feasibility of integrating NGS-based gene panel testing as part of the standard diagnostic protocol for solid tumor assessment. The high diagnostic rates achieved in this study have significant clinical implications, including improved diagnosis, prognosis, and management of patients with solid tumors.

Genomics has also long been important in the diagnosis and treatment of hematological malignancies as well. While much of the knowledge that enabled this was gained using ‘gold-standard’ cytogenetic techniques, advances in high-throughput ‘omics’ technologies have dramatically increased our knowledge of the molecular pathogenesis of these disorders. In a study by Tierens et al., investigation of the genetic profile, bone marrow morphology and immunophenotype of AML patients with biallelic disruption of *DDX41* activity identified distinct clinicopathologic hallmarks specific to patients with these alterations. These genotype-phenotype correlations in *DDX41*-related hematologic malignancies highlight the importance of biallelic alterations in the pathophysiology of this acute leukemia entity.

The genetic landscape of sarcomatoid hepatocellular carcinoma (HSC), a rare and aggressive malignancy, was investigated in a study by Jia et al. Through comprehensive genetic profiling, high mutation rates were observed in genes involved in the TP53 and DDR pathways. Furthermore, actionable mutations in genes such as *MET* fusions, *NTRK1* fusions, and *BRCA1/2* mutations were identified in HSCs indicating the genetic complexity of HSC. These findings have important implications for treatment decisions, as they shed light on the potential therapeutic options for HSC patients and emphasizes the importance of understanding the underlying genetic testing in order to develop targeted therapies for this challenging aggressive cancer.

In the context of non-small cell lung cancer (NSCLC), rapid identification of *EGFR* mutations is crucial for selecting the most appropriate treatment. The IdyllaTM system, a rapid and accurate method, was introduced for the detection of EGFR mutations (Qiu et al.). This integrated cartridge-based system provided results within 2.5 hours, enabling timely decision-making for NSCLC patients. Compared to other diagnostic techniques, the IdyllaTM system demonstrated high sensitivity, specificity, and concordance rates, making it a valuable tool for guiding fast treatment decisions in NSCLC.

Liquid biopsy has gained prominence in recent years, as it offers a minimally invasive alternative to traditional tumour tissue or bone marrow biopsies, and also allows real-time monitoring of tumor dynamics and genetic alterations. Three papers highlight its utility in clinical diagnostics to identify rare/low frequency variants, as well as epigenetic alterations. Hallermayr et al. explore this modality as a complementary approach to tissue biopsy with a combined workflow using NGS and duplex sequencing technology to identify low-frequency variants in plasma. Using this approach, they successfully identified the molecular etiology of an asymmetric overgrowth syndrome in a 10-year-old child that would have remained undetected with tissue analysis alone.

In another study, researchers demonstrated the utility of liquid biopsy and targeted NGS to detect chromosomal structural abnormalities or copy number variations (CNVs) in patients with myeloid neoplasms (Ip et al.). The high concordance rate seen when compared to cytogenetic data from corresponding bone marrow samples demonstrate the utility of liquid biopsy in specific circumstances when obtaining a bone marrow biopsy is not possible.

The third study addressed the challenges for early detection of breast cancer due to its heterogeneity and low levels of circulating tumor DNA (ctDNA) using a multimodal liquid biopsy approach called SPOT (Pham et al.). This approach simultaneously analyzed genome-wide methylation changes, copy number alterations, and 4-nucleotide oligomer end motifs in ctDNA of breast cancer patients. By combining these multiple signatures and utilizing machine learning, this model achieved enhanced accuracy in the detection of early-stage breast cancer. This approach has the potential to improve breast cancer screening and reduce false positive rates.

In conclusion, the implementation of genomic innovations in clinical cancer diagnostics has significantly advanced our understanding of the genetic landscape of tumors and improved patient care. The successful implementation of precision medicine relies on the collaborative efforts of clinical laboratory geneticists, oncologists, pathologists, and medical geneticists. These stakeholders have a responsibility to integrate these advancements into their practice and optimize the characterization of the genetic profile of various cancers, opening new avenues for precision medicine and personalized cancer care. Continued research and technological advancements in this field will further enhance our ability to diagnose, predict, and treat cancer, ultimately leading to improved patient outcomes and a more effective approach to cancer management.

## Author contributions

LS: Conceptualization, Project administration, Writing – original draft. AM: Writing – original draft, Writing – review & editing. SS: Writing – original draft, Writing – review & editing.

